# Current News

**Published:** 1898-07

**Authors:** 


### Current News.

### ALUMNI SOCIETY OF THE PHILADELPHIA DENTAL COLLEGE.

THE Annual Meeting of the Alumni Society of the Philadelphia Dental College was held at three P.M., March 31, in the college building at Eighteenth and Buttonwood Streets. President Leo Greenbaum, M.D., D.D.S., occupied the chair. Many of the alumni were present.

The Committee on Photographs reported the receipt of the photograph of the late Dr. Thomas Evans, of Paris, and appealed to the alumni in general for gifts of enlarged photographs or oil portraits of men eminent in the profession.

The Committee on Certificates of Membership reported progress.

The following papers were read and discussed. By. W. G. Chase, of Philadelphia, "Caries of the Maxilla." By Dr. W. A. Capon, of Philadelphia, "Practical Points on Porcelain Inlays." By Dr. J. A. Boland, of Philadelphia, "Experiences in Dental Practice."

The election of officers resulted as follows:

President, Professor Leo Greenbaum; Vice-Presidents, Dr. Charles E. Butler and Dr. J. W. Allen (Class of '98); Treasurer, Dr. Charles Vanderslice; Secretary, Dr. Otto E. Inglis; Corresponding Secretary, Dr. Eliza Yerkes.

Adjourned.

OTTO E. INGLIS,
*Secretary.*

### CENTRAL DENTAL ASSOCIATION OF NORTHERN NEW JERSEY.

At the Annual Meeting of the Central Dental Association of Northern New Jersey the following officers were elected for the ensuing year:

President, F. Edsall Riley, D.D.S., 474 Broad Street, Newark; Vice-President, C. S. Hardy, D.D.S., Summit; Secretary, H. S. Sutphen, D.D.S., 24 East Kinney Street, Newark; Treasurer, Charles A. Meeker, D.D.S., 29 Fulton Street, Newark.

*Executive Committee.*—F. G. Gregory, D.D.S., Chairman, 7 West Park Street, Newark; Fred. C. Barlow, D.D.S., Jersey City; C. W. Boblitzell, D.D.S., Jersey City; W. H. Pruden, D.D.S., Paterson; W. E. Truex, D.D.S., Freehold.

H. S. Sutphen,
*Secretary.*

---

### CHICAGO DENTAL SOCIETY.

List of officers of the Chicago Dental Society for 1898–99, elected at the Annual Meeting, held Tuesday evening, April 5, 1898: President, J. E. Hinkins; First Vice-President, D. C. Bacon; Second Vice-President, E. A. Royce; Recording Secretary, Elgin Ma Whinney; Corresponding Secretary, C. S. Bigelow; Treasurer, E. D. Swain; Member Board of Directors, J. G. Reid.

*Board of Censors.*—A. W. Harlan, W. V.-B. Ames, C. N. Johnson.

C. S. Bigelow,
*Corresponding Secretary.*

---

### DENTAL SOCIETY OF THE STATE OF NEW YORK.

At the Thirtieth Annual Meeting of the Dental Society of the State of New York, held on the 12th inst., the following officers were elected for the ensuing year:

President, F. Le Grand Ames, D.D.S., Albany; Vice-President, John I. Hart, D.D.S., New York; Secretary, Charles S. Butler, D.D.S., Buffalo; Treasurer, Charles W. Stainton, D.D.S., Buffalo; Correspondent, R. Ottolengui, M.D.S., New York.

Charles S. Butler,
*Secretary.*

